# Intercostal Nerve Transfer for Biceps Reinnervation in Obstetrical Brachial Plexus Palsy: A Preferred Reporting Items for Systematic Reviews and Meta-Analysis for Individual Patient Data Systematic Review using Individualized Fusion and Comparison to Supraclavicular Exploration and Nerve Grafting

**DOI:** 10.1177/18632521231211644

**Published:** 2023-12-04

**Authors:** George Abdelmalek, George Ehab Mina, Krittika Pant, Zheshi Zheng, Jasmine Mahajan, Nivetha Srinivasan, Shivani Gupta, Jasmine Shafei, Michael F Levidy, Aleksandra McGrath, Alice Chu

**Affiliations:** 1 Rutgers New Jersey Medical School, Newark, NJ, USA; 2 Department of Statistics, Rutgers University, Piscataway, NJ, USA; 3 Department of Hand Surgery, Norrland’s University Hospital, Umea, Sweden; 4 Department of Anatomy, Umea University, Umea, Sweden; 5 Division of Pediatric Orthopedics, Department of Orthopedic Surgery, Rutgers New Jersey Medical School, Newark, NJ, USA

**Keywords:** Obstetric brachial plexus palsy, nerve grafting, nerve transfer, intercostal, elbow flexion

## Abstract

**Introduction:**

The objective of this study was to search existing literature on nerve reconstruction surgery in patients with obstetric brachial plexus palsy to determine whether treatment with supraclavicular exploration and nerve grafting produced better elbow flexion outcomes compared to intercostal nerve transfer.

**Methods:**

This study was a systematic review following the Preferred Reporting Items for Systematic Reviews and Meta-Analysis for Individual Patient Data guidelines. A systematic search was conducted using multiple databases. An ordinal regression model was used to analyze the effect of using supraclavicular exploration and nerve grafting or intercostal nerve on elbow flexion with the two scores measured: elbow flexion Medical Research Council scores and Toronto active movements scale scores for elbow flexion.

**Results:**

A final patient database from 6 published articles consisted of 83 supraclavicular exploration and nerve grafting patients (73 patients with Medical Research Council and 10 patients with Toronto score) and 7 published articles which consisted of 131 intercostal nerve patients (84 patients with Medical Research Council and 47 patients with Toronto scores). Patients who underwent supraclavicular exploration and nerve grafting presented with an average Medical Research Council score of 3.9 ± 0.72 and an average Toronto score of 6.2 ± 2.2. Patients who underwent intercostal nerve transfer presented with an average Medical Research Council score of 3.9 ± 0.71 and an average Toronto score of 6.4 ± 1.2. There was no statistical difference between supraclavicular exploration and nerve grafting and intercostal nerve transfer when utilizing Medical Research Council elbow flexion scores (ordinal regression: 0.3821, standard error: 0.4590, p = 0.2551) or Toronto Active Movement Scale score for elbow flexion (ordinal regression: 0.7154, standard error: 0.8487, p = 0.2188).

**Conclusion:**

Regardless of surgical intervention utilized (supraclavicular exploration and nerve grafting or intercostal nerve transfers), patients had excellent outcomes for elbow flexion following obstetric brachial plexus palsy when utilizing Medical Research Council or Toronto scores for elbow flexion. The difference between these scores was not statistically significant.

**Type of study/Level of evidence:**

Therapeutic Study: Investigating the Result of Treatment/level III.

## Introduction

With an estimated incidence between 0.4 and 4 per 1000 live births obstetric brachial plexus palsy (OBPP) is rare but can be a debilitating injury at birth. The extent of nerve involvement is different among patients and can be categorized into the following syndromes: upper trunk (C5-C6 ± C7) and complete (C5-T1). While complete palsy presents with effects on shoulder, elbow, wrist, and hand movement, patients with upper trunk palsy most prominently have a lack of active shoulder abduction and elbow flexion.^1,2^ While most OBPP cases may result in spontaneous recovery, there are a variety of primary and secondary surgical interventions that can be used to treat OBPP to improve function of the affected limb.^3^ Primary surgical interventions include neurolysis, nerve grafting and nerve transfers.

Traditionally, primary surgery to improve elbow flexion in OBPP involves a supraclavicular incision, subsequent exploration of the brachial plexus to identify viable proximal roots and then attachment of these roots to the trunk, cord, or peripheral nerve through a nerve graft.^4,5^ While proximal nerve transfers are performed near the site of injury in the supra- or infra-clavicular fossa, distal nerve transfers are those performed beyond the brachial plexus zone and near the neuromuscular junction.

Proximal nerve transfers usually include diagnosing the lesion via brachial plexus exploration and dissection, followed by surgical intervention. These procedures have been well studied and produce excellent outcome. This operation presents with some disadvantages including longer recovery times and greater technical demands as compared to nerve transfers.^6^

Unlike proximal nerve transfers, distal nerve transfers are performed away from the site of injury and closer to the target muscle, do not require nerve grafts, result in shorter surgery times and, skills wise, are within grasp for a surgeon without high volume exposure to OBPP cases due to decreased technical demand and subjectivity of the procedure. Although there are clear benefits of distal nerve transfers, these are at the expense of full donor nerve function and post-operative complications such as respiratory failure.^6^ The distal procedure of intercostal nerve (ICN) transfer for reconstruction of the musculocutaneous nerve (MCN) has been used for adult brachial plexus injury. More recent studies have shown it to be effective in obstetric brachial palsy patients, with 70%–90% of patients achieving greater than or equal than Medical Research Council (MRC) M3 strength of biceps.^7,8^

Elbow flexion is critical to a child’s development and to many activities of daily living, from eating to buttoning a shirt. OBPP involving the C5-C6 nerve roots and global palsy without recovery of C5-C6 frequently results in reduced to absent elbow flexion.^9^ Thus, the reconstructive strategies currently employed in improving elbow flexion in OBPP patients are of considerable interest. Given that there has been no conclusive determination as to whether one procedure may be superior to the other, there is a significant need to analyze the current data.

The aim of this study was to analyze all available literature and assess whether supraclavicular exploration with nerve grafting or ICN transfer is more effective in improving elbow flexion utilizing a variety of outcome measures.

## Methods

### Literature search

This study was conducted under Preferred Reporting Items for Systematic Reviews and Meta-Analysis for Individual Patient Data (PRISMA-IPD) guidelines. First, a systematic search of the literature was conducted using PubMed, Cochrane, Web of Science, and the Cumulative Index to Nursing and Allied Health Literature (CINAHL) databases. Specific search terms including “brachial plexus,” “injury,” “palsy,” “nerve plexus,” “upper plexus,” “pediatric,” and “surgery” were used. The complete collection of Boolean searches is provided in Supplemental Appendix A. From the initial set of articles, duplicates were removed, followed by an abstract and full-text screening. In these screenings to build the preliminary database, English text studies on brachial plexus surgery in pediatric patients were identified. The exclusion criteria for these studies were as follows: (1) studies that were not full text; (2) studies classified as commentaries, review papers, or editorials; (3) studies that were non-human or had less than three participants; (4) studies which had full texts that were inaccessible. The full-length texts were accessed online. For completion, the references of all selected articles were cross-checked. If these articles were not previously included and fulfilled the inclusion/exclusion criteria, they were included in the database.

From this preliminary database, articles were then screened for relevance to this study’s specific objective. Only studies which investigated patients who received ICN transfers or treatment with supraclavicular exploration and nerve grafting (SENG) were included. Studies with ICN nerve transfers and SENG were then subdivided into groups based upon the type of outcome measures used to evaluate elbow flexion. These included the Medical Research Council (MRC) Scale for Muscle Strength (elbow flexors) and elbow flexion Toronto Active Movement Scale (Table 1).

**Table 1.** ICN and SENG studies.

Study	Average age	Mean outcome for each outcome measure	Time of evaluation	General findings
Meyer^10^	Mean age at surgery = 4.2 months (range = 3–6 months)	Evaluated MRC and Mallet scores for shoulder and elbow function	Followed 16 infants, 11 treated conservatively and 5 who underwent surgical intervention	Observed those patients treated operatively had return in function of grafted areas with one having prior excellent nerve action potentials across neuroma
Ghanghurde et al.^11^	Mena age at time of surgery was 5.8 months (range = 3–12 months)	Shoulder: mean Mallet score observed was 15 Elbow: flexion strength at final follow-up was 4+ in all patients Hand: preserved hand function	Patients were assessed at a mean age of 2.8 years	Oberlin transfers produced excellent outcomes in regard to restoration of elbow flexion with all patients presenting at least a 4+ (holds test position against moderate to strong pressure)
Chuang et al.^12^	Average age of infant period = 4.9 months (range = 2–11 months) 31 cases Average beyond the infant period = 19.2 months (range = 12–30 months) 1 case—age 1 year	MRC Elbow flexion: easy M = 3 (good), difficult M = 2 (fair), impossible M < 2 (poor) 31/44 ICN-MCN under 1 year if age elbow flexion score: 20 good, 7 fair, 4 poor 1/10 ICN-MCN beyond 1 year of age elbow flexion score: M0 → M2 = fair	Study analyzed surgical explorations on 78 infant obstetrical brachial plexus palsy patients from 1992 to 1999. Of the procedures that involved ICN—all came from rupture injury associated with root avulsion (44/78 of the total exploration patients) totaling 31/44 ICN-MCN for elbow function Each patient had a 4-year follow-up	Study analyzed the timing, early versus delayed, and treatment strategies for patients with isolated rupture and rupture with root avulsion. Study found that early nerve surgery when indicated is still the treatment of choice for BPP, with no difference in elbow function of surgery between 2 and 11 months Hand palsy is a major indicator for early surgery <3 months ICN transfer to MCN are significantly better in obstetrical BPP compared with adults
Noaman et al.^13^	Average age at time of operation was 16 months (range = 11–24 months)	Seven children with OBPP indicated for surgical intervention due to absent elbow flexion with active shoulder abduction 5/7 achieved ≥ M3 elbow flexion 2/7 achieved < M3	The average follow-up was 19 months (range = 13–30 months)	Oberlin procedure is a good option for C5-C6 nerve root avulsion in patients who present late with no biceps function but adequate shoulder function
Xu et al.^14^	Mean age of nerve transfer and grafting group was 4.5 months (range = 3–6 months)	Evaluated shoulder function with Mallet Evaluated supraspinatus, deltoid, and biceps strength with MRC MRC biceps: M5: 1/10 M4:4/10 M3: 3/10 M2: 2/10	The follow-up period on average was 44.3 months in the nerve transfer and grafting group	The nerve transfer and grafting group achieved greater recovery of should and elbow range of motion and had more favorable outcome in comparison to neurolysis and conservative treatments
Pondaag and Malessy^15^	Mean age at surgery for ICN-MCN = 5.5 months 17/42 cases Mean age at surgery for MPN-MCN = 6 months 25/42 cases	Bicep strength evaluated using MRC grading system—dichotomized: >/=MRC 3 versus <MRC 3 ICN-MCN 14/17 = success of reinnervation with three failures (<MRC 3) MPN-MCN 23/25 = success with two failures Total results: 1/42 complete failure 4/42 had some contraction, but not against gravity (MRC1–2) 9/42—MRC 3 28/42—MRC 4	Consecutive series of 33 patients at single institution between 1995–2002 used ICN, C5 and C6 nerve roots	Study primarily investigated the difference between ICN versus MPN to MCN in order to reinnervate bicep muscle. Although the results in the MPN–MCN group were slightly better than in the ICN–MCN group, possibly due to more axons, larger size, and exclusive motor function, there was no statistically significant difference between the two groups. Suggestion is that MPN-MCN is the first choice for treatment
Kawabata et al.^8^	Average age at surgery = 5.8 months (range = 3–14 months) 12 pts. < 5 months 14 pts. 5–8 months 3 pts. 8–11 months 1 pt 14 months	Power of biceps muscle using the standard British Research council Muscle Movement Scale—M0 to M4 M0—no contraction M1—trace of contraction M2—active flexion more than 90 degrees without gravity M3—active flexion more than 90 against gravity M4—active movement against gravity and resistance, no limited ROM M5—not elicited in child Graded M4 in 26/31 = 84% of patients 12 patients who underwent surgery < 5 months—M4 = 100% in biceps power Grade M3 in 3/31 Grade M2 in 2/31	Study analyzed 31 neurotizations of MCN using ICN in 30 patients, 1 bilateral, with Birth-related brachial plexus injuries. Conducted on complete avulsion of the cervical nerve roots No mention of time span Mean follow-up period was 5.2 years	Study stated that ICN transfer is a viable method of neurotization of the MCN in birth palsy with root avulsions. Outcomes are better in infants than adults, with minimal donor site complications (no sensory deficits, no respiratory dysfunction) There was no relationship between type of paralysis and post-operative outcome
Pondaag and Malessy^16^	Mean age at surgery = 4.4 months (range = 3–8 months)	At least 4/33 received ICN–MCN transfers. Those surgeries performed after 2002, undetermined elbow function follow-up MRC—medical research council grading system used for elbow flexion MRC grade 4—¾ cases MRC grade 3—¼ cases	Retrospective analysis of 33 surgical reconstruction cases over a 10–year period—1995–2004 for those with flail arm + lack of muscle contraction to direct nerve stimulation 4/33 cases received an ICN-MCN for elbow flexion Follow-up in cases (before 2002) = 50 months	Study focused mostly on the analysis of hand function following nerve grafting and transfer for those with a flail arm. Primary goal is restoration of hand function, secondary goal is elbow flexion, tertiary goal is shoulder function. As elbow flexion was only a secondary goal, the ICN–MCN procedure/outcomes was not focused on In many of the cases, elbow flexion was obtained not via use of ICN but using C5/C6 nerve roots to the superior trunk
Luo et al.^7^	Mean age at surgery = 5 months (range = 3–11 months) All patients lacked biceps function initially	Bicep strength evaluated using British MRC grading (M0–5) Improvement of biceps noted at median 9.3 months for ADUT versus 7.8 for MCN ICN-MCN 11/12: M4 strength 1/12: M3 ICN-ADUT 6/12: M4 4/12: M3 2/12: M2 4 ICNs used: 8/14: M4 4/14: M3 2/14: M2 3 ICNs used: 8/9: M4 1/9: M3 2 ICNs used: 1/1: M4	Retrospective analysis of 24 patients who underwent ICN-MCN, 12 cases, and ICN-AD of upper trunk, 12 cases, reconstruction from March 2003 to October 2005 Treatment of root avulsion Follow-up = average 53 months	Study investigated whether surgical results were directly proportional to the number of transferred ICNs and whether the anterior division of upper trunk was an appropriate site of coaptation of ICNs. Results showed lack of consensus along with other studies on the number of ICNs used and outcome. Lack of power in this test to reach a statistical significance but use of four ICNs may be redundant as it obtains the similar result of M4 strength as two to three ICNs. ICN-MCN proved to be better than ICN-ADUT, showing that the distal attachment may be better in terms of biceps flexion. No respiratory problems found, no problems in sensation via pin stimulation, all patients could flex independent of breathing
Lara et al.^17^	A total of 48 patients were operated upon between 3 and 6 months of age. Two patients had delayed nerve transfers due to phrenic nerve deficits	At 5 years post-operatively, M3 scores were noted in all 48 patients who received undelayed nerve transfers which improved to M4 in patients. In the two patients who had delayed nerve transfers, the biceps did not recover	A mean follow-up of 10 years with a range of 8–20 years	This study mostly examined respiratory complications associated with intercostal nerve harvest—of which there were no such complications. ICN transfers proved to be a reliable option for elbow flexion restoration following OBPP when undelayed
Sénès et al.^18^	Two procedures performed from ICN → MCN—out of 32 patients Eight procedures performed for elbow flexion: XI → MCN, Oberlin (ulnar nerve), ICN → MCN Mean 31.3 months ± 19.4 months (for the entire 32 patients)	Elbow function was scored using: scale of Nerve Injury Committee of British Medical Research, modified by Mackinnon and Dellon Results reported as: good, fair, bad ICN → MCN transfer reported 1 Good (>M4) rating and 1 fair (M4–/M3+) rating Reported similar results for Oberlin versus ICN	Spanning from January 2005 to January 2011—32 partially recovered patients that received late surgery—5 months to 6.6 years. Follow-up 27.5 months mean	Study looked at the surgical outcomes for repair of OBPP injuries that did not meet the early surgery criteria. These were therefore late surgical repairs. With regard to MCN repair, study stated that if possible, first option should be the use of ulnar nerve fascicle transfer over the ICN Results showed that late nerve surgical procedures can be effective in children affected by OBPP, up until the age of 6
Al-Qattan and Al-Kharfy^19^	A retrospective chart review of 10 patients with presentations of OBPP with lack of active elbow flexion against gravity. Age at time of surgery ranged from 13 to 19 months	Assessment of elbow flexion using Toronto Score 0 = 1/10 patients 1 = 0/10 2 = 0/10 3 = 0/10 4 = 0/10 5 = 0/10 6 = 1/10 7 = 8/10	Follow-up time ranged from 1 to 2 years	In late presenting patients, median nerve utilization for biceps reinnervation is an excellent option with nearly all patients obtaining full motion at the final post-operative period
El-Gammal et al.^20^	Age at surgery = 41 months mean (range = 18 months–4 years) for all 19 cases 5 ICN transfers versus 6 Oberlin transfers 11 total elbow flexion cases	Assessment of motor recovery using Toronto Active Movement Scale: poor < 4, fair = 5, good = 6, and full = 7 Poor and fair = unsatisfactory Good and full = satisfactory Active elbow flexion—increased from 2.7 degrees preop → 91.8 degrees post-op = average Average grade of 0.4 → 4.7 in the Toronto scale ICN transfer had 104 average active motion gain versus 76.7 for Oberlin Power gain of 4.6 versus 4 for ICN versus Oberlin	Nineteen cases presented after age 1 with poor recovery of elbow/hand function Follow-up 50 months average. Unknown time frame of patients studied	Study looked at various different late presenting OBPP cases and surgical treatment for elbow flexion, extension, and hand function. No statistical difference between ICN versus Oberlin transfer in regard to range of motion, power grade, or onset of contraction. Oberlin preferred when C7 intact, however, concludes that best and most consistent results of delayed selective neurotization are obtained by neurotizing the biceps muscle by the ICN or in select cases by the FCU fascicle of the ulnar nerve
Semaya et al.^21^	Mean age at surgery = 40.6 months (range = 24–65 months)	Seven-point Toronto scale developed by Curtis et al. and Clarke and Curtis for motor power Preop elbow flexion: Grade 1 = 2 patients Grade 2 = 12 patients Grade 3 = 2 patients Mean grade = 2.0 Final follow-up: Grade 3–1 patient Grade 4–3 patients Grade 6–9 patients Grade 7–3 patients Mean grade = 5.625 Mean active range of motion of elbow against gravity—increased from 38 degrees → 96.8 degrees	Sixteen cases with OBPP Follow-up for mean of 51.7 months	This study specifically looked at the phenomenon of cocontraction of the biceps and triceps due to spontaneously recovered OBPP that have undergone cross-innervation. ICN → MCN was used to manage the cocontraction Improvement was noted in elbow flexion in all cases except 1, triceps function was preserved, with improvement of cocontraction with bonus in separation of motion of the shoulder and elbow joints due to misdirected axons to MCN Recommendation for procedure in children 2 years or older with cases of cocontraction

ICN: intercostal nerve; SENG: supraclavicular exploration and nerve grafting; MRC: Medical Research Council; OBPP: obstetric brachial plexus palsy; MPN: medial pectoral nerve; MCN: musculocutaneous nerve; ROM: range of motion; ADUT: anterior division of the upper trunk; AD: anterior division; XI: 11th cranial nerve (accessory nerve); FCU: flexor carpi ulnaris; BPP: brachial plexus palsy.

### Data extraction

Data extracted from articles in the preliminary database included number of patients, patient characteristics, follow-up, outcomes following surgery, and information about any secondary procedures. Data extraction was conducted by all the authors. The authors of articles that did not have comprehensive data were contacted for additional information. If these authors did not respond or could not provide additional data, these studies were excluded from the present analysis.

### Statistical methods

We employed an ordinal regression model to analyze the effect of using SENG or ICN on elbow flexion with the two scores measured: elbow flexion MRC and elbow flexion Toronto Active Movement Scale. We choose ordinal regression because it is designed for order response categorical variable and allows for the independent variable (SENG/ICN method in our case) to be either continuous or discrete. We used a logit link function which is the most widely used in statistical application literature, and the model is formulated as: log(*Pr*(*Score*≤*j*)*Pr*(*Score*>*j*)) = β0*j*;+β1*Method*+ɛ.

## Results

**Figure 1.**
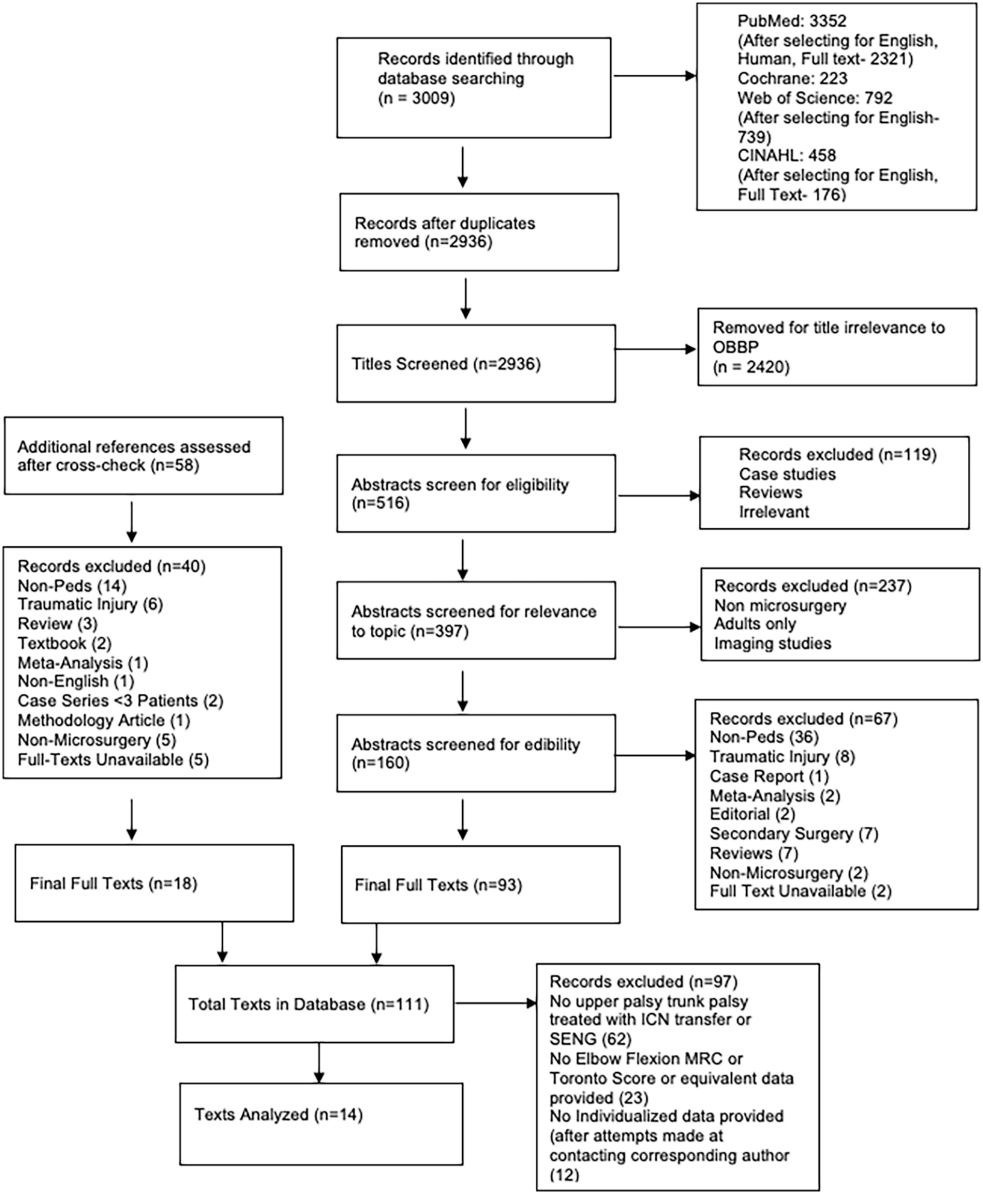
PRISMA workflow.

**Figure 2.**
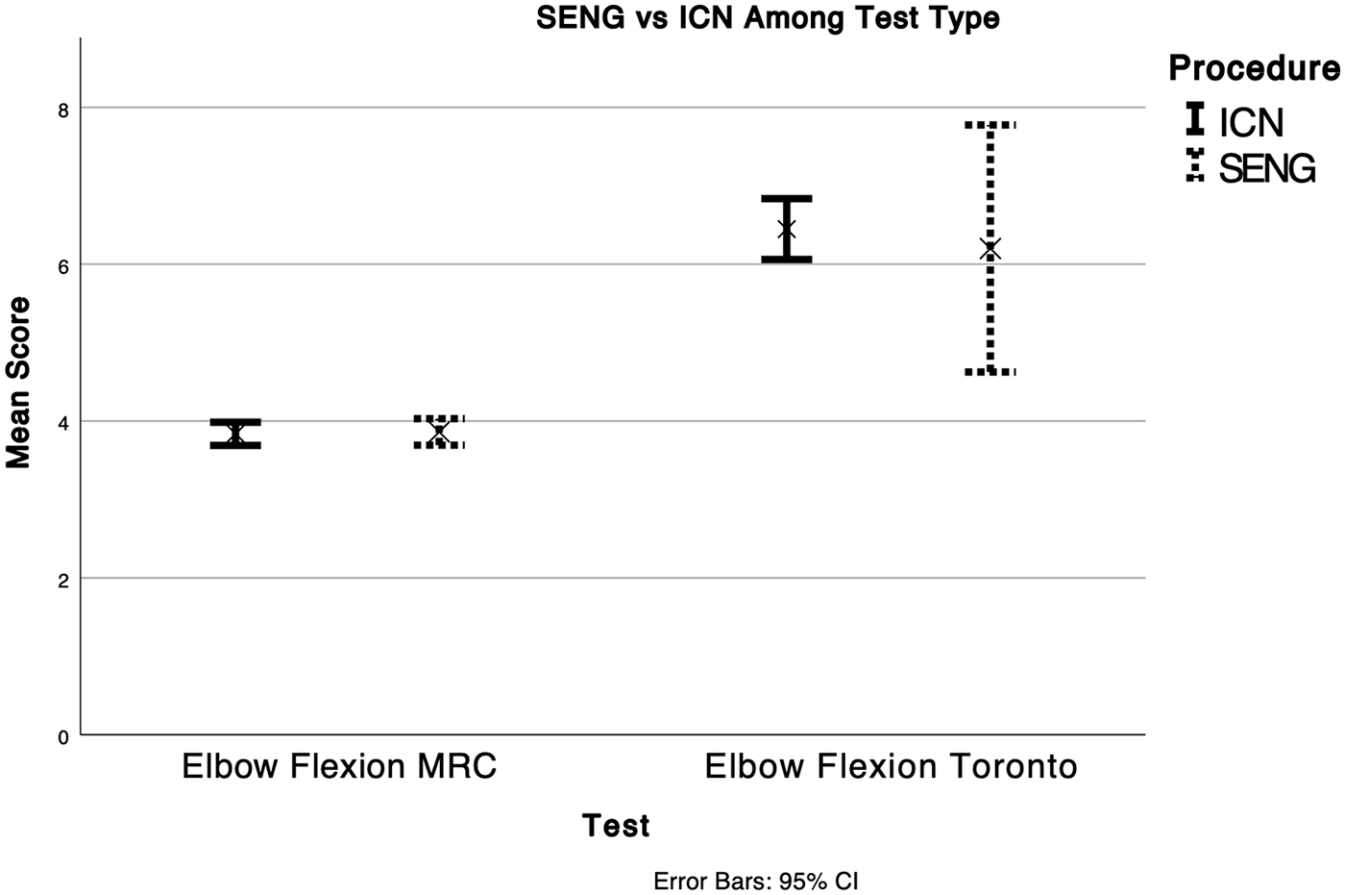
SENG versus ICN among test type (elbow flexion MRC, elbow flexion Toronto).

The PRISMA-IPD workflow used to identify studies is shown in Figure 1. Initially, 2936 texts were identified using the search terms. After a title screening, texts that were irrelevant to pediatric brachial plexus palsies, case reports, traumatic injuries, meta-analyses, editorials, secondary surgeries, reviews, non-microsurgical operations, and unavailable full texts were excluded, resulting in an initial index of 93 full texts. An additional 18 texts were identified by cross-references. This produced a preliminary database of 111 texts. Afterwards, texts were screened for relevancy to SENG and ICN transfers. This produced 49 texts. Following this, the authors screened the published texts for results reported (elbow flexion MRC and elbow flexion Toronto scores) (Figure 2). In addition, the authors filtered out articles that reported grouped results or non-standardized scores. For example, some authors chose to report scores of as MRC scores 4 and 5 as excellent, 2 and 3 as adequate, and 1 as unsuccessful. Attempts were made to reach out to these authors for a more detailed report of patient outcomes; however, this was unsuccessful in most instances. A final patient database from six published articles consisted of 83 SENG patients (73 patients with MRC and 10 patients with Toronto score) and seven published articles which consisted of 131 ICN patients (84 patients with MRC and 47 patients with Toronto scores).

Data gathered from six texts of patients undergoing SENG present an average MRC score of 3.9 ± 0.72.^10–15^ Data gathered from five texts of patients undergoing ICN transfers present an average MRC score of 3.9 ± 0.71.^7,8,16–18^ Data gathered from one text of patients undergoing SENG present an average Toronto score of 6.2 ± 2.2.^19^ Data gathered from two texts of patients undergoing ICN transfers present an average Toronto score of 6.4 ± 1.2.^20,21^ The regression value between SENG and ICN transfers when comparing MRC scores is MRC scores is 0.3821 (standard error 0.4590, p = 0.2551). The regression value between SENG and ICN transfers when comparing Toronto scores is 0.7154 (standard error 0.8487, p = 0.2188). There is no statistical significance between SENG and ICN transfers when utilizing MRC or Toronto scores.

## Discussion

Currently, there is little guidance for the use of different surgical procedures for patients undergoing repair for OBPP. Treatments for patients with upper nerve palsies emphasize the restoration of active shoulder abduction and elbow flexion. Traditionally, the use of SENG has produced favorable outcomes for patients. However, newer procedures that include the use of ICN transfers have not been extensively compared to proximal surgeries for elbow flexion in the pediatric population. We have found that both procedures result in excellent patient outcomes as a function of elbow flexion measured using the MRC score.

The use of SENG provides surgeons and patients with advantages for repair. This surgical procedure has been the standard for brachial plexus lesions in adults and children. Multiple studies have shown that this procedure has provided patients with a safe and effective treatment option for upper palsies. Furthermore, the surgical window allows for not only repair but also diagnosis and visualization of the lesion. This allows repair of multiple deformities and injuries that may be present in patients during one operation. While these procedures have been well studied and produce excellent outcomes, this operation presents with some disadvantages including the morbidity of longer dissections in an already traumatized area, longer operative times, and the necessity of subjective factors such as visual assessment of lesions in continuity and relying on intraoperative nerve action potentials.^6,22^ In addition, SENG procedure is not appropriate in all patients, especially in more extensive injuries with poor quality of proximal donors accompanied by root avulsion.

Distal nerve surgeries can provide patients with an improved post-surgical recovery experience as patients tend to have a faster recovery period. Furthermore, there is strong evidence that in the adult population, intra-plexus dual nerve transfers provided better outcomes for shoulder and elbow functions in patients with traumatic upper plexus palsy compared to traditional nerve grafting. However, this procedure does present with some technical and clinical difficulty. While distal transfers spare some operative morbidity due to their distance from the site of injury, this same distance can be a drawback, preventing diagnostic visualization of the affecting lesion.^4^ This coupled with potentially decreased donor nerve function and lower Mallet scores can call into question the value of ICN procedures compared to SENG,^6^ taking into account that pros and cons of both procedures can only be debated for a group of patients where the both options exist, as opposed to patients with poor proximal donors where SENG cannot be performed. Furthermore, complications of ICN transfers have been explored and include pleural tears, wound infections, pleural effusions, acute respiratory distress syndrome, and wound seromas.^23^ Because of the lack of long term studies, it is unclear the total effect of ICN transfers in the long term such as chest deformity or influence on ventilation at an older age. With the literature showing cases of deformity at 20 years post–operatively, there should be hesitancy to make ICN transfer a first-line treatment for pediatric populations.^17^

Others have expressed the concern of impaired respiratory function following ICN transfer with concomitant phrenic nerve transection. This concern may be unfounded as the literature has shown comparable pulmonary function in adults after ICN transfer. In a study of 42 adult patients undergoing phrenic nerve transfers and ICN transfers, it was found that there was no significant difference in phrenic nerve transfers and multiple ICN transfers compared to phrenic nerve transfers alone.^24^ In addition, other studies concluded that 10 adult patients undergoing simultaneous phrenic and ICN transfers did not produce clinically evident respiratory dysfunction post-operatively.^25^ To our knowledge, there are no studies describing these complications in pediatric patients.

While conducting this study, many of the shortcomings regarding literature data presentation were highlighted. Some of these shortcomings can be attributed to a lack of studies containing children undergoing ICN transfers. In addition, a lack of standardization when compiling results contributed to the limited number of studies that can be compared. Data are often grouped differently between authors as some choose to report results as a function of time, procedure, location, or final outcome. This lack of standardization makes it difficult for surgeons to compare treatment options when choosing the operations that are best for their patients. Furthermore, very few authors provide individual data on patient outcomes. This can make it difficult to compare results and identify differences between selection criteria and functional results between different sets of literature. These issues are especially prevalent in the literature surrounding the treatment and assessment of brachial plexus injuries. Previous attempts have been made to find a consensus on how to report data using the international Plexus Outcome Study Group (iPLUTO) project.^26^ Some of the recommendations include the use of a data set and timing protocol when providing data in all scientific papers.

There are several limitations to this study. Limited sample sizes and inability to personally and empirically assess each patient hindered our ability to provide concrete insights and recommendations. Furthermore, nerve grafting to the anterior division of the upper trunk and ICN transfer to the MCN are not always utilized for different types and severities of injury. This prevented direct comparison between the two surgical options and can introduce bias. The use of ICN transfers for obstetric brachial plexus palsies continues to be an area of unknown for clinicians. Comprehensive and comparative studies for this treatment option are sparse. For physicians and surgeons to be fully equipped to help their patients, there needs to be a standardized, systemic approach for result presentation.

## Conclusion

Both SENG and ICN transfers produce favorable outcomes for patients suffering from OBPP. There are a plethora of functional outcomes used to evaluate shoulder, elbow, and wrist function following surgical intervention. Elbow flexion is of particular interest as it is crucial in motor skill development of an infant. In this study, SENG and ICN transfers produced equivalent outcomes when utilizing MRC elbow flexion scores or Toronto active movement scale scores for elbow flexion.

## Supplemental Material

sj-docx-1-cho-10.1177_18632521231211644 – Supplemental material for Intercostal Nerve Transfer for Biceps Reinnervation in Obstetrical Brachial Plexus Palsy: A Preferred Reporting Items for Systematic Reviews and Meta-Analysis for Individual Patient Data Systematic Review using Individualized Fusion and Comparison to Supraclavicular Exploration and Nerve Grafting Click here for additional data file. Supplemental material, sj-docx-1-cho-10.1177_18632521231211644 for Intercostal Nerve Transfer for Biceps Reinnervation in Obstetrical Brachial Plexus Palsy: A Preferred Reporting Items for Systematic Reviews and Meta-Analysis for Individual Patient Data Systematic Review using Individualized Fusion and Comparison to Supraclavicular Exploration and Nerve Grafting by George Abdelmalek, George Ehab Mina, Krittika Pant, Zheshi Zheng, Jasmine Mahajan, Nivetha Srinivasan, Shivani Gupta, Jasmine Shafei, Michael F Levidy, Aleksandra McGrath and Alice Chu in Journal of Children’s Orthopaedics

sj-pdf-2-cho-10.1177_18632521231211644 – Supplemental material for Intercostal Nerve Transfer for Biceps Reinnervation in Obstetrical Brachial Plexus Palsy: A Preferred Reporting Items for Systematic Reviews and Meta-Analysis for Individual Patient Data Systematic Review using Individualized Fusion and Comparison to Supraclavicular Exploration and Nerve Grafting Click here for additional data file. Supplemental material, sj-pdf-2-cho-10.1177_18632521231211644 for Intercostal Nerve Transfer for Biceps Reinnervation in Obstetrical Brachial Plexus Palsy: A Preferred Reporting Items for Systematic Reviews and Meta-Analysis for Individual Patient Data Systematic Review using Individualized Fusion and Comparison to Supraclavicular Exploration and Nerve Grafting by George Abdelmalek, George Ehab Mina, Krittika Pant, Zheshi Zheng, Jasmine Mahajan, Nivetha Srinivasan, Shivani Gupta, Jasmine Shafei, Michael F Levidy, Aleksandra McGrath and Alice Chu in Journal of Children’s Orthopaedics

## References

[1] Hale HB Bae DS Waters PM. Current concepts in the management of brachial plexus birth palsy. J Hand Surg Am 2010; 35(2): 322–331. 20141905 10.1016/j.jhsa.2009.11.026

[2] O’Berry P Brown M Phillips L , et al. Obstetrical brachial plexus palsy. Curr Probl Pediatr Adolesc Health Care 2017; 47(7): 151–155. 28709767 10.1016/j.cppeds.2017.06.003

[3] Soucacos PN Vekris MD Kostas J , et al. Secondary reconstructive procedures in obstetrical brachial plexus palsy: forearm, wrist, and hand deformities. Semin Plast Surg 2005; 19(1): 96–102.

[4] Garg R Merrell GA Hillstrom HJ , et al. Comparison of nerve transfers and nerve grafting for traumatic upper plexus palsy: a systematic review and analysis. J Bone Joint Surg Am 2011; 93(9): 819–829. 21543672 10.2106/JBJS.I.01602

[5] Terzis JK Papakonstantinou K. Surgical treatment of obstetrical brachial plexus paralysis: the Norfolk Experience. Semin Plast Surg 2004; 18(4): 359–375.

[6] McGrath AM Lu JC Chang TN , et al. Proximal versus distal nerve transfer for biceps reinnervation—a comparative study in a rat’s brachial plexus injury model. Plast Reconstr Surg Glob Open 2016; 4(12): e1130. 28293499 10.1097/GOX.0000000000001130 PMC5222644

[7] Luo PB Chen L Zhou CH , et al. Results of intercostal nerve transfer to the musculocutaneous nerve in brachial plexus birth palsy. J Pediatr Orthop 2011; 31(8): 884–888. 22101669 10.1097/BPO.0b013e318230a783

[8] Kawabata H Shibata T Matsui Y , et al. Use of intercostal nerves for neurotization of the musculocutaneous nerve in infants with birth-related brachial plexus palsy. J Neurosurg 2001; 94(3): 386–391. 11235940 10.3171/jns.2001.94.3.0386

[9] Terzis JK Kokkalis ZT. Secondary procedures for elbow flexion restoration in late obstetric brachial plexus palsy. Hand 2010; 5(2): 125–134. 19430848 10.1007/s11552-009-9198-3 PMC2880668

[10] Meyer RD. Treatment of adult and obstetrical brachial plexus injuries. Orthopedics 1986; 9(6): 899–903. 3725694 10.3928/0147-7447-19860601-17

[bibr11-18632521231211644] Ghanghurde BA Mehta R Ladkat KM , et al. Distal transfers as a primary treatment in obstetric brachial plexus palsy: a series of 20 cases. J Hand Surg Eur Vol 2016; 41(8): 875–881. 27543083 10.1177/1753193416663887

[bibr12-18632521231211644] Chuang DC Mardini S Ma HS. Surgical strategy for infant obstetrical brachial plexus palsy: experiences at Chang Gung Memorial Hospital. Plast Reconstr Surg 2005; 116(1): 132–142. Discussion 143–144. 15988259 10.1097/01.prs.0000169936.19073.b4

[bibr13-18632521231211644] Noaman HH Shiha AE Bahm J. Oberlin’s ulnar nerve transfer to the biceps motor nerve in obstetric brachial plexus palsy: indications, and good and bad results. Microsurgery 2004; 24(3): 182–187. 15160375 10.1002/micr.20037

[bibr14-18632521231211644] Xu J Cheng X Gu Y. Different methods and results in the treatment of obstetrical brachial plexus palsy. J Reconstr Microsurg 2000; 16(6): 417–420. Discussion 420–422. 10993086 10.1055/s-2006-947147

[15] Pondaag W Malessy MJ. Intercostal and pectoral nerve transfers to re-innervate the biceps muscle in obstetric brachial plexus lesions. J Hand Surg Eur Vol 2014; 39(6): 647–652. 23940103 10.1177/1753193413501588

[16] Pondaag W Malessy MJ. Recovery of hand function following nerve grafting and transfer in obstetric brachial plexus lesions. J Neurosurg 2006; 105(Suppl. 1): 33–40. 16871868 10.3171/ped.2006.105.1.33

[bibr17-18632521231211644] Lara AM Bhatia A Correa JC , et al. Intercostal nerve transfers to the musculocutaneous–a reliable nerve transfer for restoration of elbow flexion in birth-related brachial plexus injuries. Indian J Plast Surg 2020; 53(2): 254–259. 32884191 10.1055/s-0040-1716186 PMC7458836

[18] Sénès F Catena N Sénès J. Nerve transfer in delayed obstetrical palsy repair. J Brachial Plex Peripher Nerve Inj 2015; 10(1): e2–e14. 10.1055/s-0035-1549367 PMC5023088 27917233

[19] Al-Qattan MM Al-Kharfy TM. Median nerve to biceps nerve transfer to restore elbow flexion in obstetric brachial plexus palsy. Biomed Res Int 2014; 2014: 854084. 24511548 10.1155/2014/854084 PMC3910468

[20] El-Gammal TA El-Sayed A Kotb MM , et al. Delayed selective neurotization for restoration of elbow and hand functions in late presenting obstetrical brachial plexus palsy. J Reconstr Microsurg 2014; 30(4): 271–274. 24696398 10.1055/s-0033-1357280

[21] Semaya AE El-Nakeeb R Hasan M , et al. Intercostal nerve transfer in management of biceps and triceps cocontraction in spontaneously recovered obstetric brachial plexus palsy. Ann Plast Surg 2019; 83(4): 447–451. 31524740 10.1097/SAP.0000000000002075

[22] Heise CO Siqueira MG Martins RS , et al. Distal nerve transfer versus supraclavicular nerve grafting: comparison of elbow flexion outcome in neonatal brachial plexus palsy with C5–C7 involvement. Childs Nerv Syst 2017; 33(9): 1571–1574. 28647810 10.1007/s00381-017-3492-0

[23] Kovachevich R Kircher MF Wood CM , et al. Complications of intercostal nerve transfer for brachial plexus reconstruction. J Hand Surg Am 2010; 35(12): 1995–2000. 21095076 10.1016/j.jhsa.2010.09.013

[24] Zheng MX Qiu YQ Xu WD , et al. Long-term observation of respiratory function after unilateral phrenic nerve and multiple intercostal nerve transfer for avulsed brachial plexus injury. Neurosurgery 2012; 70(4): 796–801. 22426043 10.1227/NEU.0b013e3181f74139

[25] Malungpaishope K Leechavengvongs S Ratchawatana P , et al. Simultaneous phrenic and intercostal nerves transfer for elbow flexion and extension in total brachial plexus root avulsion injury. J Hand Surg Asian Pac Vol 2018; 23(4): 496–500. 30428802 10.1142/S2424835518500480

[26] Pondaag W Malessy MJA . Outcome assessment for brachial plexus birth injury. J Orthop Res 2018; 36(9): 2533–2541. 29566312 10.1002/jor.23901 PMC6175006

