# Supplementary material for: Intercostal Nerve Transfer for Biceps Reinnervation in Obstetrical Brachial Plexus Palsy: A Preferred Reporting Items for Systematic Reviews and Meta-Analysis for Individual Patient Data Systematic Review using Individualized Fusion and Comparison to Supraclavicular Exploration and Nerve Grafting
Source: J Child Orthop. 2023 Dec 4;18(1):54–63. doi: 10.1177/18632521231211644 (PMC10859120; doi:10.1177/18632521231211644)
Supplement: sj-docx-1-cho-10.1177_18632521231211644 – Supplemental material for Intercostal Nerve Transfer for Biceps Reinnervation in Obstetrical Brachial Plexus Palsy: A Preferred Reporting Items for Systematic Reviews and Meta-Analysis for Individual Patient Data Systematic Review using Individualized Fusion [file sj-docx-1-cho-10.1177_18632521231211644.docx]

**Supplemental material**

((brachial plexus pals*[MeSH] OR brachial plexus pals*[tiab] OR brachial plexus injury[MeSH] OR brachial plexus injury[MeSH] OR brachial plexus injury[tiab] OR nerve plexus injury[MeSH] OR nerve plexus injury[tiab] OR brachial nerve injury[MeSH] OR brachial nerve injury[tiab] OR[MeSH] OR brachial plexus surgery[tiab] OR brachial nerves injury[MeSH] OR brachial nerves injury[tiab] OR tbpi[MeSH] OR tbpi[tiab] or traumatic[MeSH] OR traumatic bpi[tiab]) AND (infant[MeSH] OR child[MeSH] OR adolescent[MeSH] OR children[MeSH] OR child[tiab] OR child*[tiab] OR adolescent[MeSH] OR adolescent[tiab] OR infant[tiab] OR baby[tiab] OR young[tiab] OR youth[tiab] OR kid[tiab] OR kids[tiab] OR pediatric[MeSH] OR pediatric[tiab] OR pediatrics[MeSH] OR pediatrics[tiab]) AND (surg*[MeSH] OR surg*[tiab] OR oper*[MeSH] OR oper*[tiab]))

((brachial plexus pals* OR brachial plexus pals* OR brachial plexus injury OR brachial plexus injury OR nerve plexus injury OR brachial nerve injury OR brachial plexus surgery OR brachial nerves injury OR tbpi or traumatic bpi) AND (infant OR child OR adolescent OR children OR child OR child* OR adolescent OR adolescent OR infant OR baby OR young OR youth OR kid OR kids OR pediatric OR pediatrics) AND (surg* OR oper*))
